# A Damage Model of Concrete including Hysteretic Effect under Cyclic Loading

**DOI:** 10.3390/ma15145062

**Published:** 2022-07-20

**Authors:** Zhi Liu, Li Zhang, Lanhao Zhao, Zihan Wu, Bowen Guo

**Affiliations:** 1 Jiangxi Academy of Water Science and Engineering, Nanchang 330029, China; lz19880701@163.com; 2 Jiangxi Provincial Technology Innovation Center for Ecological Water Engineering in Poyang Lake Basin, Nanchang 330095, China; 3 College of Water Conservancy and Hydropower Engineering, Hohai University, Nanjing 210098, China; leewit@hhu.edu.cn; 4 School of Infrastructure Engineering, Nanchang University, Nanchang 330031, China; 3231237548a@gmail.com; 5 Yellow River Institute of Hydraulic Research, Zhengzhou 450003, China; guobowen21@126.com

**Keywords:** damage model, concrete, cyclic loading, hysteresis effect, seismic analysis

## Abstract

A novel damage model for concrete has been developed, which can reflect the complex hysteresis phenomena of concrete under cyclic loading, as well as other nonlinear behaviors such as stress softening, stiffness degradation, and irreversible deformation. The model cleverly transforms the complex multiaxial stress state into a uniaxial state by equivalent strain, with few computational parameters and simple mathematical expression. The uniaxial tensile and compressive stress–strain curves matching the actual characteristics are used to accommodate the high asymmetry of concrete in tension and compression, respectively. Meanwhile, an unloading path and a reloading path that can reflect the hysteresis effect under cyclic loading of concrete are established, in which the adopted expressions for the loading and unloading characteristic points do not depend on the shape of the curve. The proposed model has a concise form that can be easily implemented and also shows strong generality and flexibility. Finally, the reliability and correctness of the model are verified by comparing the numerical results with the three-point bending beam test, cyclic loading test, and a seismic damage simulation of the Koyna gravity dam.

## 1. Introduction

Concrete, as the most versatile construction material, has significant nonlinear characteristics due to the microcracks that accompany its formation. Especially under unconventional loadings such as earthquakes, their high destructive and unique unpredictability will make the nonlinear characteristics of concrete structures even more intense. The simulation of the nonlinear characteristics of concrete is usually based on fracture mechanics, plasticity mechanics, and continuum damage mechanics. Fracture mechanics focus on the local nonlinearity caused by macrocracks, which contradicts the distribution pattern of microcracks [1,2,3,4,5,6]. The crack evolution process is also different from that of metallic materials based on crystal slip or dislocation, so the plastic mechanics is difficult to apply [7,8,9,10] for the material of concrete. Continuous damage mechanics, on the other hand, captures the nonlinear behavior of concrete by introducing damage variables to characterize the dispersive evolution of cracks [11,12]. Some scholars have proposed a class of models called the elastic damage model, which can reflect the softening process of concrete and the phenomenon of stiffness degradation after unloading. However, the irreversible deformation after unloading is ignored, making it unsuitable for cyclic loading [13,14,15,16,17,18]. Accordingly, the elastoplastic damage model is widely used to capture the behavior of concrete under cyclic loading for its ability to account for irreversible deformation after unloading [19,20,21,22,23]. For the traditional elastoplastic damage model, the unloading and reloading paths are expressed linearly, and the damage remains constant during this process, which cannot fully reflect the true damage pattern under cyclic loading. The stiffness degradation and stress redistribution caused by damage accumulation and energy dissipation will inevitably affect the subsequent change process of the nonlinear performance of the concrete. The accumulation of damage and energy dissipation from the continuous unloading and reloading process will form an obvious hysteresis effect. At present, there are few pieces of research on the hysteretic rules under concrete cyclic loading. Scholars mostly derive mathematical formulas based on experimental data to simplify the hysteretic behavior under cyclic compressive loading [24], and a few also study hysteretic rules under cyclic tensile loading [25,26,27]. Konstantinidis [28] performed statistics on the current constitutive model of concrete under cyclic compressive loads. Aslani [29] and Guo [30] summarized the characteristics of concrete hysteretic behavior. Traditional damage models usually simplify the hysteresis effect to a linear expression. This assumption of describing nonlinear phenomena in a linear form cannot fully reflect the damage accumulation process under cyclic loading. The delayed damage accumulation cannot feedback the stress transfer of the degraded part in time. It will inevitably affect the subsequent simulation results. Li [31] combined the uniaxial stress–strain curve with the hysteretic model proposed by Yassin [32] and established a hysteretic constitutive model for nonlinear analysis under cyclic loading. However, the reloading curve of the hysteretic rule in the model will return to the unloading point, which can represent the stiffness degradation but cannot accurately describe the damage accumulation process. The applicability of the actual engineering needs to be further improved. Based on the existing four-parameter damage model of concrete, this paper combines the loading and unloading characteristic points and the loading and unloading path in the hysteretic rules to construct a four-parameter damage model considering the hysteretic effect under cyclic loading. The model contains complex nonlinear characteristics such as tension and compression anomalies, stiffness degradation, strength softening, irreversible plastic deformation, and hysteresis effects during the evolution of concrete under cyclic loading. Furthermore, through the concrete uniaxial cyclic load test and the earthquake damage simulation of the Koyna gravity dam, the correctness of the model in solving the nonlinear problem is verified.

## 2. Fundamental Governing Equations

### 2.1. Concrete Four-Parameter Damage Model

As a simplification and modification of the Ottosen criterion, Hsieh-Ting-Chen’s four-parameter failure criterion [33] based on stress space exhibits a good convergence in numerical calculations. On the basis of the criterion, Li [34] established a four-parameter failure criterion based on strain space:

(1) $$F\left(I_{1}^{\prime },J_{2}^{\prime },\varepsilon_{0}\right)=A\frac{J_{2}^{\prime }}{\varepsilon_{0}}+B\sqrt{J_{2}^{\prime }}+C\varepsilon_{1}+DI_{1}^{\prime }=0$$

where, $I_{1}^{\prime }=\varepsilon_{ii}(i=1,2,3)$ is the first invariant of the strain tensor, $J_{2}^{\prime }=e_{ij}e_{ij}/2(i,j=1,2,3)$ is the second invariant of the strain tensor, $\varepsilon_{1}=\frac{2}{\sqrt{3}}\sqrt{J_{2}^{\prime }}\sin(\theta +\frac{2}{3}\pi )+\frac{1}{3}I_{1}^{\prime }$ is the maximum principal strain, $\theta =\frac{1}{3}\arcsin(-\frac{3\sqrt{3}J_{3}^{\prime }}{2\sqrt{{J_{2}^{\prime }}^{3}}})$ $\varepsilon_{0}=\frac{f_{t}}{E}$ is the peak strain of the material, $J_{3}^{\prime }=e_{ij}e_{jk}e_{ki}(i,j,k=1,2,3)$ is the third invariant of strain deviation, the four parameters A, B, C, and D are constants, which are obtained jointly by four characteristic strength values suggested in the literature [35].

It is assumed that the four-parameter failure criterion is applicable in the strain-softening section, and the four parameters, A, B, C, and D, remain unchanged. The form is the same as the Equation (1), which is replaced by the equivalent strain:

(2) $$\varepsilon^{*}=A\frac{J_{2}^{\prime }}{\varepsilon^{*}}+B\sqrt{J_{2}^{\prime }}+C\varepsilon_{1}+DI_{1}^{\prime }$$

where, $\varepsilon_{1}=\frac{2}{\sqrt{3}}\sqrt{J_{2}^{\prime }}\sin(\theta +\frac{2}{3}\pi )+\frac{1}{3}I_{1}^{\prime }$ is the maximum principal strain, $I_{1}^{\prime }=(\varepsilon_{1}+\varepsilon_{2}+\varepsilon_{3})/3$ is the first invariant of the strain tensor, $J_{2}^{\prime }=\frac{1}{2}\left[{\left(\varepsilon_{1}-\varepsilon_{m}\right)}^{2}+{\left(\varepsilon_{2}-\varepsilon_{m}\right)}^{2}+{\left(\varepsilon_{3}-\varepsilon_{m}\right)}^{2}\right]$ is the second invariant of strain deviation, $\theta =-\frac{3\sqrt{3}J_{3}^{\prime }}{2\sqrt{J_{2}^{\prime }}}$, $J_{3}^{\prime }=\varepsilon_{1}\varepsilon_{2}\varepsilon_{3}$ is the third invariant of strain deviation, $\varepsilon_{1},\varepsilon_{2},\varepsilon_{3}$ are the three principal strains of *x, y, z*.

The four parameters, A, B, C, and D, are the same as those used in the failure criterion. The equivalent strain under a multiaxial stress state can be obtained by solving Equation (2) and taking into account that $\varepsilon^{*}\geq 0$:

(3) $$\varepsilon^{*}=\frac{(B\sqrt{J_{2}^{\prime }}+C\varepsilon_{1}+DI_{1}^{\prime })+\sqrt{{\left(B\sqrt{J_{2}^{\prime }}+C\varepsilon_{1}+DI_{1}^{\prime }\right)}^{2}+4AJ_{2}^{\prime }}}{2}$$

The above formula is simple and clear and can transform complex multiaxial problems into a simple uniaxial one in equivalent space. The model has been fully theoretically verified by previous work [34,36,37].

### 2.2. Stress Unloading Residual Strain Value

The irreversible deformation, that is, residual strain, will occur when the concrete is unloaded after reaching the softening phase. It is usually ignored since the empirical formula fitted by the test results is difficult to capture the critical value. However, when the load duration is long enough or the number of loading and unloading times is sufficient, it will eventually have a deviation, which is manifested $E_{c}⩽0$ after exceeding the critical value.

Many researchers have proposed different residual strain formulations for different constitutive models [38,39,40]. In this paper, the formula suggested by Vecchio and Palermo [25] is selected:

(4) $$\varepsilon_{p}=\varepsilon_{r}\left(0.166{\left(\frac{\varepsilon_{un}}{\varepsilon_{r}}\right)}^{2}+0.132\left(\frac{\varepsilon_{un}}{\varepsilon_{r}}\right)\right)$$

where, $\varepsilon_{p}$ is the plastic residual strain, $\varepsilon_{r}$ is the peak tensile or compressive strength corresponding to strain, $\varepsilon_{un}$ is the strain at the unloading point.

**Figure 1:**
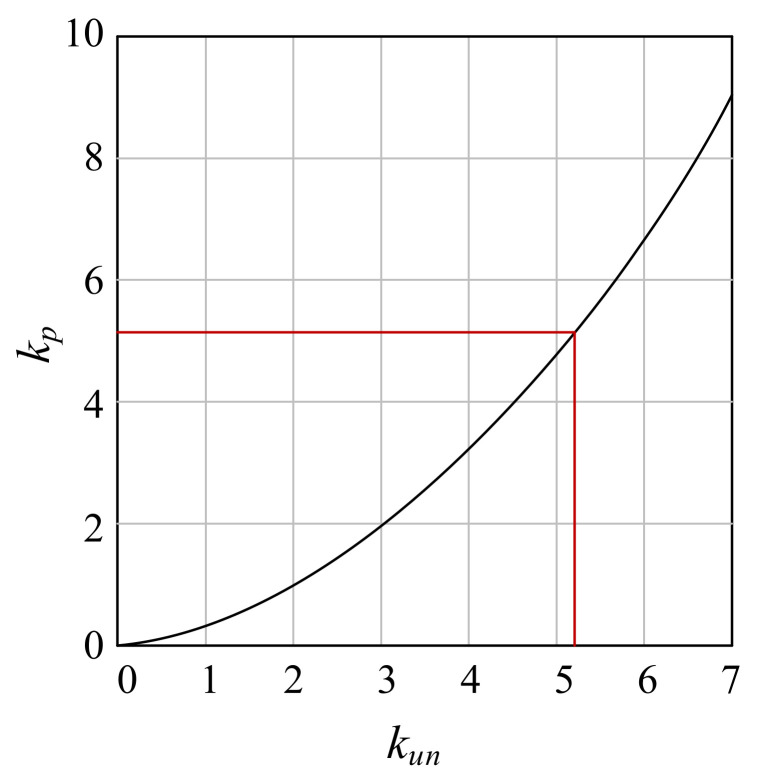
The critical value of residual strain.

Let $k_{p}=\frac{\varepsilon_{p}}{\varepsilon_{r}}$, $k_{un}=\frac{\varepsilon_{un}}{\varepsilon_{r}}$, and compare $k_{p}$ with $k_{un}$, as shown in Figure 1, when $k_{un}\approx 5.23$, $k_{p}=k_{un}$, that is, unloading stiffness E=0. So take the critical value $k_{un}=4.5$, when $k_{un}⩾4.5$, $\varepsilon_{p}=0.85\varepsilon_{un}$.

### 2.3. Uniaxial Stress–Strain Curve

In this paper, the stress–strain curve proposed by Guo [30] was chosen. Considering the anisotropy of concrete in tension and compression, the uniaxial stress–strain curves were selected separately according to the tensile and compressive states. For the convenience of description, the stress and the strain are expressed as a relative value:

(5) $$x=\frac{\varepsilon^{*}}{\varepsilon_{0}},y=\frac{\sigma^{*}}{f_{t}}$$

where, $\varepsilon^{*}$ is equivalent strain, $\sigma^{*}$ is equivalent stress, $f_{t}$ is the peak strength, $\varepsilon_{0}$ is the strain corresponding to peak strength.

Whether in tension or compression, the stress–strain curve consists of two phases: the elastic phase and the softening phase.

**Figure 2:**
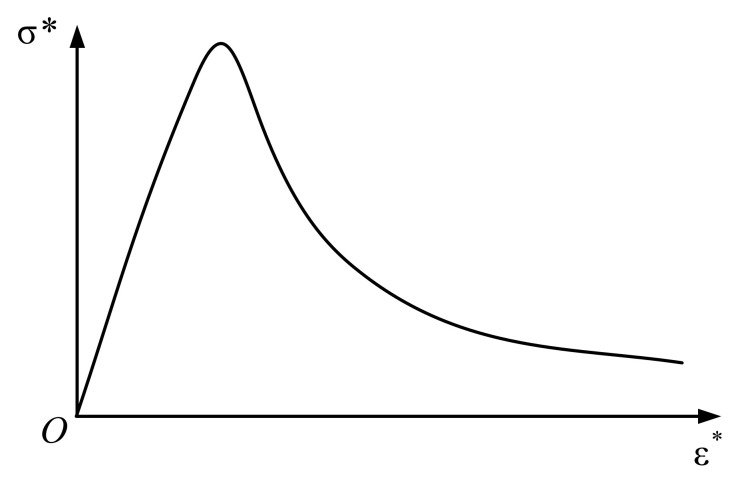
Uniaxial tensile stress–strain curve.

As is shown in Figure 2, for uniaxial tension, the elastic section $\left(x<1\right)$ can be expressed as:

(6) $$y=1.2x-0.2x^{6}$$

The softening phase $\left(x>1\right)$ is:

(7) $$y=\frac{x}{a{\left(x-1\right)}^{1.7}+x}$$

where, the factor 1.2 in the elastic phase is the ratio of the initial modulus to the cutline modulus at the peak point, the factor *a* in softening phase is obtained from the empirical formula, $a=0.312f_{t}^{2}$, which follows the change of tensile strength.

**Figure 3:**
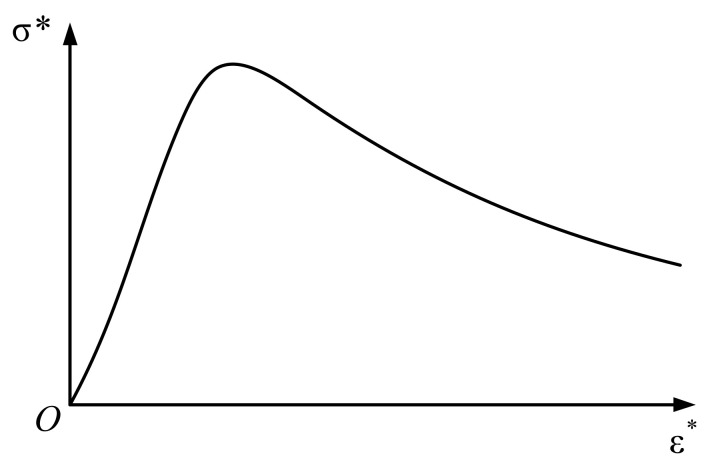
Uniaxial compressive stress–strain curve.

As is shown in Figure 3, for uniaxial compression, the elastic section $\left(x\leq 1\right)$ can be expressed as:

(8) $$y=\beta x+(3-2\beta )x^{2}+(\beta -2)x^{3}$$

The softening phase $\left(x\geq 1\right)$ is:

(9) $$y=\frac{x}{\gamma {\left(x-1\right)}^{2}+x}$$

where, the factor β in the elastic phase is the ratio of the initial modulus to the cutline modulus at the peak point, 1.5≤β≤3, the factor γ in softening phase is 0≤γ≤∞, when γ=0, y=1, the softening phase is plastic, when γ=∞, y=0, the softening phase is brittle.

### 2.4. Damage Variable Values

According to the strain equivalence principle, the stress-strain relationship of concrete can be expressed as:

(10) $$\sigma =\sigma (\varepsilon ,\varepsilon_{0},D)=\varepsilon \cdot E_{0}(1-D)$$

Based on the above uniaxial stress-strain curves, the damage values corresponding to each stage can be deduced as follows:

(11) $$D=\left\{\begin{array}{cc} 0 & x\leq 1\\ 1-\frac{\sigma }{E_{0}\varepsilon } & x\geq 1 \end{array}\right.$$

When it is a multiaxial state, the real stress and strain need to be replaced by the equivalent stress and strain:

(12) $$D=\left\{\begin{array}{cc} 0 & x\leq 1\\ 1-\frac{\sigma^{*}}{E_{0}\varepsilon^{*}} & x\geq 1 \end{array}\right.$$

When under cyclic loading, the residual strain will be taken into account:

(13) $$D=\left\{\begin{array}{cc} 0 & x\leq 1\\ 1-\frac{\sigma^{*}}{E_{0}(\varepsilon^{*}-\varepsilon_{p}^{*})} & x\geq 1 \end{array}\right.$$

## 3. Implementation Process

Through the observations of the concrete cyclic loading experiments, it was found that the stress–strain response under cyclic loading depends on the load history. At the same time, the hysteresis effect is not limited to complete unloading and complete reloading. There are partial unloading cycles with incomplete unloading and partial reloading with incomplete reloading. Otter [41] established a set of mathematical, empirical formulas based on load history by observing test data to derive the loading and unloading characteristic points under cyclic loading. The formula has good applicability to plain concrete and reinforced concrete and does not depend on the shape of the stress–strain skeleton line.

### 3.1. Complete Loading and Unloading Cycle

**Figure 4:**
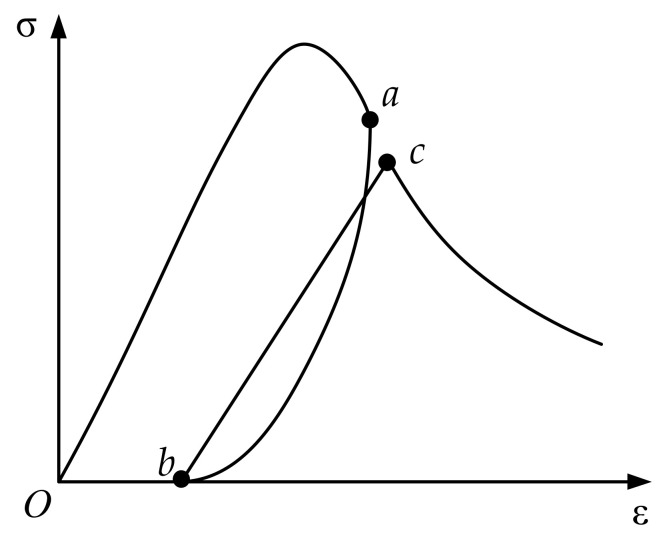
Complete unloading and reloading of the damage model (The letters *a*, *b*, *c* are points on the curve of the complete loading and unloading cycle).

The complete loading and unloading cycle is shown in Figure 4. The hysteretic cycle consists of the unloading path *ab* and the reloading path *bc*. The degree of damage accumulation during the hysteresis effect is reflected in the model as the strain on the skeleton line develops from the unloading point *a* to the reloading point *c*.

According to the unloading strain $\varepsilon_{un}$ at unloading point ***a***, the strain $\varepsilon_{re}$ at the reloading point ***c*** will be obtained:

(14) $$\frac{\varepsilon_{re}}{\varepsilon_{r}}=\frac{\varepsilon_{un}}{\varepsilon_{r}}+k_{r}$$

where, $k_{r}$ is the reloading coefficient, and the recommended value is 0.1.

The curvature of the unloading curve in the hysteresis rule reflects the change in the stiffness, and the secant modulus changes continuously from large to small. The reloading curve can be simplified to linear, during which the secant modulus remains constant, and the damage value does not change during the period [27]. The complete unloading curve adopts the empirical expression of test fitting by Sima [42], in which the damage variable is included, which can reflect the damage accumulation in the unloading process:

(15) $$\sigma =\xi_{1}e^{\xi_{2}\left(1-\frac{\varepsilon -\varepsilon_{p}}{\varepsilon_{un}-\varepsilon_{p}}\right)}E_{c}\left(\varepsilon -\varepsilon_{p}\right)$$

(16) $$\sigma =\frac{\varepsilon -\varepsilon_{p}}{\varepsilon_{re}-\varepsilon_{p}}\sigma_{re}$$

where, $\xi_{1}=\frac{r\left(1-d_{un}\right)}{r-1}$, $r=\frac{\varepsilon_{un}}{\varepsilon_{p}}$, $\xi_{2}=Ln\left[\frac{R\left(1-d_{un}\right)\left(r-1\right)}{r}\right]$, $d_{un}$ is the damage value at the unloading point, $\sigma_{re}$ is stress value at the reloading point, $R=\frac{E_{p}}{E_{c}}$, $E_{p}$ is the secant modulus when completely unloaded.

The secant modulus will remain constant during the reloading stage, and the damage value at the residual strain point is the same as the damage value at the reloading point. Therefore, the cumulative change in damage caused by unloading is $d_{re}-d_{un}$, and the damage variable during unloading is:

(17) $$d=d_{un}+\frac{d_{re}-d_{un}}{\varepsilon_{p}-\varepsilon_{un}}\left(\varepsilon -\varepsilon_{un}\right)$$

when unloading to the residual strain point, $d=d_{re}$.

### 3.2. Partial Reload Cycle

**Figure 5:**
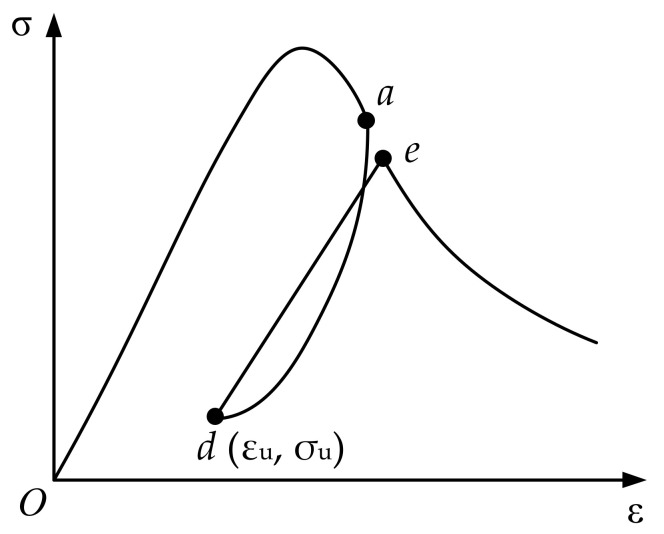
Local reloading of the damage model: the letters *a*, *d*, *e* are points on the curve of the partial reload cycle).

The partial reloading cycle in the hysteresis rule is shown in Figure 5. The hysteretic cycle consists of the unloading path *ad* and the reloading path *de*. At this time, the load is not completely unloaded to the residual strain point *b*, and the corresponding reloading point *e* is different from the reloading point *c* in the complete loading and unloading cycle. The strain value will be the interpolation between point *a* and point *c*:

(18) $$\varepsilon_{rx}=\varepsilon_{un}+\left(\varepsilon_{re}-\varepsilon_{un}\right){\left(\frac{\sigma_{un}-\sigma_{u}}{\sigma_{un}}\right)}^{n_{pu}}$$

where, $\varepsilon_{rx}$ is the strain value at the reloading point ***e***, $\sigma_{u}$ is the stress value at the lowest point ***d*** of local unloading, $n_{pu}$ is an interpolation parameter, and the recommended value is 8 after test fitting and sensitivity analysis.

The partial reload formula after unloading is the same as that for complete unloading:

(19) $$\sigma =\frac{\varepsilon -\varepsilon_{u}}{\varepsilon_{rx}-\varepsilon_{u}}\sigma_{rx}$$

where, $\varepsilon_{u}$ is the strain value at the lowest point ***d*** of local unloading, $\sigma_{rx}$ is the stress value at the reload point *e*.

### 3.3. Partial Unloading Cycle

**Figure 6:**
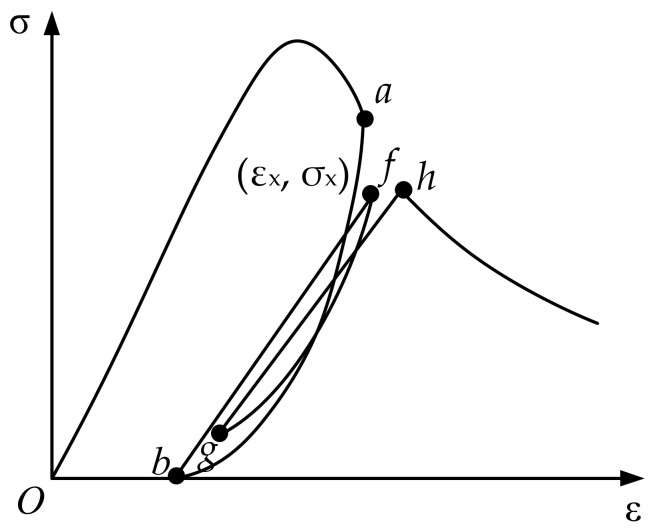
Local unloading of the damage model: the letters *a*, *b*, *f*, *g*, *h* are points on the curve of the partial unloading cycle.

The partial unloading cycle in the hysteresis rule is shown in Figure 6. The hysteretic cycle is composed of the unloading path *fg* and the reloading path *gh*. In the figure, the strain value of the unloading point corresponding to the highest point *f* of the local loading will be the interpolation between point *a* and point *c*:

(20) $$\varepsilon_{ux}=\varepsilon_{un}+\left(\varepsilon_{re}-\varepsilon_{un}\right){\left(\frac{\sigma_{x}-\sigma_{u}}{\sigma_{re}-\sigma_{u}}\right)}^{n_{pr}}$$

where, $\varepsilon_{ux}$ is the strain value at the unloading point corresponding to point ***f***, $\sigma_{x}$ is the stress value at point ***f***, $n_{pr}$ is an interpolation parameter, and the recommended value is 8.

The unloading path in the partial unloading cycle is similar to the full loading and unloading cycle:

(21) $$\sigma =\eta_{1}e^{\eta_{2}\left(1-\frac{\varepsilon -\varepsilon_{p}}{\varepsilon_{x}-\varepsilon_{p}}\right)}E\left(\varepsilon -\varepsilon_{p}\right)$$

where, $\eta_{1}=\frac{\sigma_{x}}{E\varepsilon_{x}\left(1-r_{1}\right)}$, $\eta_{2}=Ln\left[\frac{R_{1}}{\eta_{1}}\right]$, $r_{1}=\frac{\varepsilon_{x}}{\varepsilon_{p}}$, $R_{1}=\frac{E_{p}}{E_{c}}$, $\varepsilon_{p}$ is the residual strain value corresponding to $\varepsilon_{ux}$.

### 3.4. Numerical Realization of the Model

The proposed model has been embedded in finite element software GEHOMadrid through Fortran90. Using the subscripts *n* and *n* − 1 to indicate the relationship between the variable value and the time step. The specific implementation process is as follows:

1. Calculating the equivalent strain $\varepsilon_{n}^{*}$ by the Equation (3);
2. Judging the tensile and compressive state by $I_{1}^{\prime }$ to adopt different stress–strain curves;
3. Determining the state of the element at the current time step. In this paper, the states of the elements can be divided into five situations: (a) the loading state of the skeleton line, denoted as S=1; (b) the unloading state of the skeleton line, denoted as S=2; (c) the reload state, denoted as S=3; (d) the partial unloading state, denoted as S=4; (e) the pull-compression conversion state, denoted as S=5
  - When $\varepsilon_{n}^{*}\geq \varepsilon_{n-1}^{*}=\varepsilon_{\max}^{*}$, $S_{n}=1$, and computed $\varepsilon_{re}^{*}$;
  - When $\varepsilon_{p}^{*}\leq \varepsilon_{n}^{*}\leq \varepsilon_{n-1}^{*}\leq \varepsilon_{\max}^{*}$, and $S_{n-1}=1$ or $S_{n-1}=2$, computed $S_{n}=2$, $\varepsilon_{u}^{*}=\varepsilon_{n}^{*}$;
  - When $\varepsilon_{p}^{*}\leq \varepsilon_{n-1}^{*}\leq \varepsilon_{n}^{*}\leq \varepsilon_{re}^{*}$, and $S_{n-1}\neq 1$, computed $S_{n}=3$ $\varepsilon_{x}^{*}=\varepsilon_{n}^{*}$;
  - When $\varepsilon_{p}^{*}\leq \varepsilon_{n}^{*}\leq \varepsilon_{n-1}^{*}\leq \varepsilon_{x}^{*}<\varepsilon_{\max}^{*}$, and $S_{n-1}=3$ or $S_{n-1}=4$, computed $S_{n}=4$, $\varepsilon_{u}^{*}=\varepsilon_{n}^{*}$;
  - When $\varepsilon_{n}^{*}\leq \varepsilon_{n-1}^{*}$, and $\varepsilon_{n}^{*}\leq \varepsilon_{p}^{*}$ or $\varepsilon_{n}^{*}\geq \varepsilon_{n-1}^{*}$, and $S_{n-1}=5$, computed $S_{n}=5$.
4. Calculating the equivalent $\sigma_{n}^{*}$ and the damage value $d_{n}$, so that the true stress can be obtained.

## 4. Example Verification

In this section, a series of classical tests were performed on concrete specimens to validate the model proposed in this paper, including the three-point bending beam test, uniaxial cyclic tensile test, the uniaxial cyclic compressive test, uniaxial reciprocating test, and dynamic damage process of the Konya gravity dam. Some of the material parameters in the tests are set to be fixed, as listed in Table 1.

### 4.1. Three-Point Bending Beam

**Figure 7:**
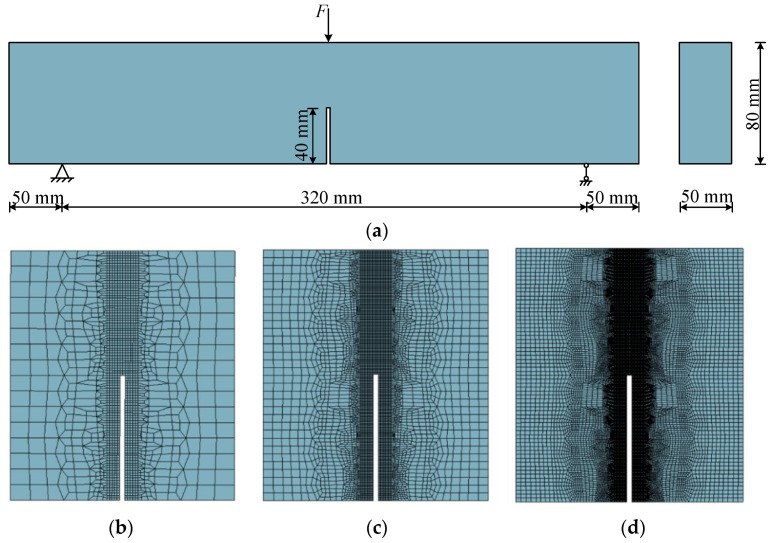
The three-point bending beam test: (**a**) dimensions and boundary conditions; (**b**) Coarse mesh; (**c**) Middle mesh; (**d**) Fine mesh.

To demonstrate the performance of the model under complex stress states, the three-point bending beam test unfolded by Toumi [43] was selected. A two-dimensional finite element model was established based on the test model shown in Figure 7. Three different meshes (Coarse mesh, Middle mesh, and Fine mesh) were conducted to examine mesh independence in terms of crack formation, and all of the meshes consisted of four-node quadrilateral elements. The bottom left pivot point of the model was constrained in both directions, the right pivot point was constrained normally, and the displacement was loaded in a graded manner at the top-center point position.

**Figure 8:**
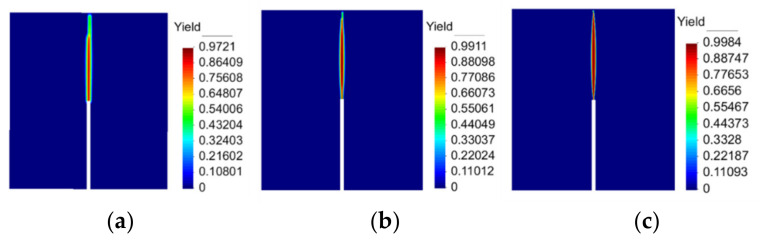
Distribution of concrete damage by different meshes: (**a**) Coarse mesh; (**b**) Middle mesh; (**c**) Fine mesh.

The distribution of the damaged area by different meshes during the continuous loading process is shown in Figure 8. The dimensions and boundary conditions result in a stress concentration at the center of the bottom of the specimen. As the prefabricated cracks cannot bear the concentrated tensile stress, the stress will be transferred from the bottom of the specimen to the other end along the prefabricated crack path. For the coarse mesh, the cracks stop propagating first before the upper edge of the beam because the elements are too large to penetrate. As the mesh becomes progressively smaller, the path of damage becomes clearer and culminates in a penetration crack in the fine mesh.

**Figure 9:**
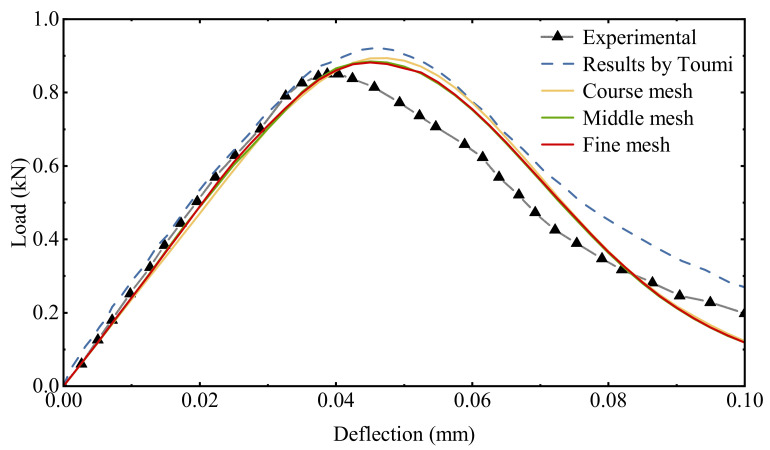
Comparison of the load-deflection curve between test and numerical results.

The load–deflection curve calculated by different meshes is presented in Figure 9. As the mesh gets larger, the reaction force gradually becomes higher. The peak value of the load–deflection curve obtained from the different meshes are all between the results of the experiment and the reference model. The trends of the curves obtained from the three meshes are generally consistent, and the differences are acceptable.

Overall, the numerical results in this section show a clear distribution pattern of damage evolution, which can fit well with the experimental data and the simulation results of reference and can verify the accuracy and applicability of the proposed model for complex stress states. The effect of the mesh size on the specimen load–deflection curve is minor.

### 4.2. Uniaxial Cyclic Tensile Loading

**Figure 10:**
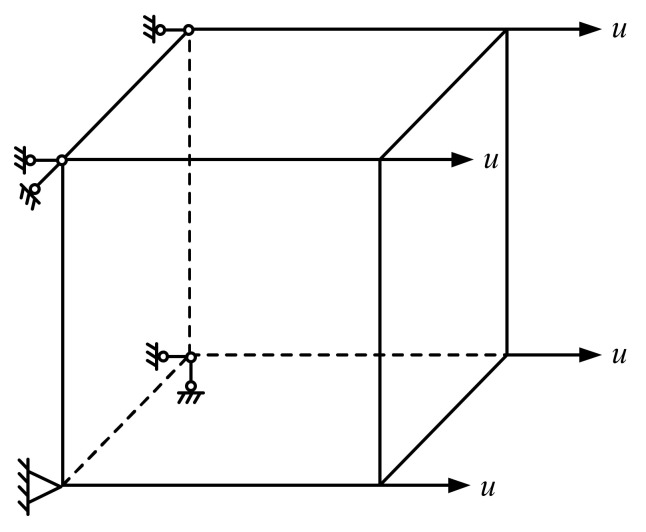
Schematic diagram of finite element model.

Based on the cyclic tensile test carried out by Gopalaratnam [44], a three-dimensional eight-node element model was established to verify the concrete damage model in this paper. As is shown in Figure 10, normal constraints were applied to the nodes on the left and bottom sides, and the nodes on the right were subjected to strain grading loading according to the test data.

**Figure 11:**
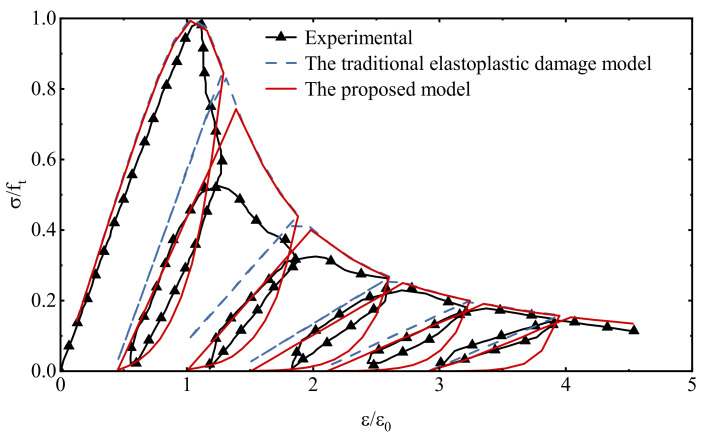
Comparison of stress–strain relationship under uniaxial cyclic tensile loading.

From Figure 11, it shows that the model in this paper can better reflect the hysteresis effect of the softening section of concrete, especially it fits well with the behavior characteristic points in the hysteresis loop, that is, the lowest point of unloading and the starting point of reloading of each hysteresis loop, usually these two points are the real data collection points for experimental observation.

**Figure 12:**
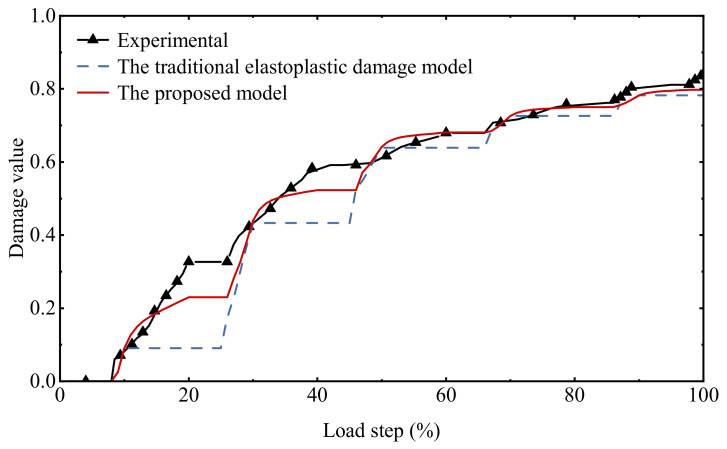
Comparison of damage history under uniaxial cyclic tensile loading.

As is shown in Figure 12, the damage history of different models and test data under cyclic tensile loading is compared. Since the test belongs to the staged loading and unloading, the data will overlap at the characteristic value of the staged loading and unloading. However, because the hysteresis effect is not considered and the damage accumulation during the unloading stage is ignored, there will be a big difference during the damage accumulation process. In the actual engineering case, the structure will be Stress redistribution occurs with the evolution of damage, and ignoring the damage accumulation during the unloading period will cause the subsequent simulation results to be quite different. In the actual engineering case, the structure will have stress redistribution as the damage evolves and will cause the subsequent simulation results to be quite different.

### 4.3. Uniaxial Cyclic Compressive Loading

In this section, the uniaxial cyclic compression test carried out by Okamoto [45] was selected, and the model and constraint schematic is referred to in Figure 10.

**Figure 13:**
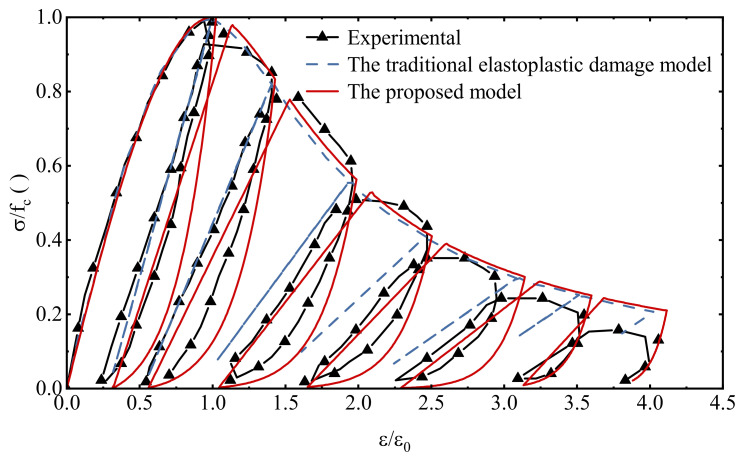
Comparison of stress−strain relationship under uniaxial cyclic compressive loading.

**Figure 14:**
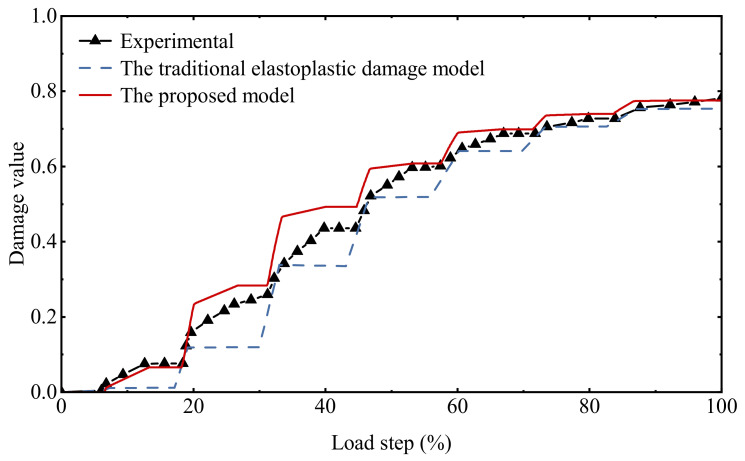
Comparison of damage history under uniaxial cyclic compressive loading.

As can be seen from Figure 13, the proposed model is reliable in describing the stress–strain relationship under uniaxial cyclic compressive loading, in which the hysteresis effect in the cyclic loading and unloading process is consistent with the experimental data, which is much better than the simulation results of the traditional elastoplastic damage model. A comparison of the damage history under cyclic compression loading is given in Figure 14. The damage accumulation process at each stage is fully reflected. Since the expressions of the hysteresis characteristic parameters in this model are mainly established based on cyclic compression tests, the simulation results fit the test data to a higher degree compared to that under cyclic tensile loading.

### 4.4. Uniaxial Reciprocating Loading

Under reciprocating loading, the material will inevitably face the problem of conversion between tension and compression, which is an indispensable part of the stress–strain curve. The purpose of this section is to validate the complete description of this model for the concrete stress–strain curve. The calculation model, material parameters, and boundary conditions are referred to as a uniaxial cyclic loading test.

**Figure 15:**
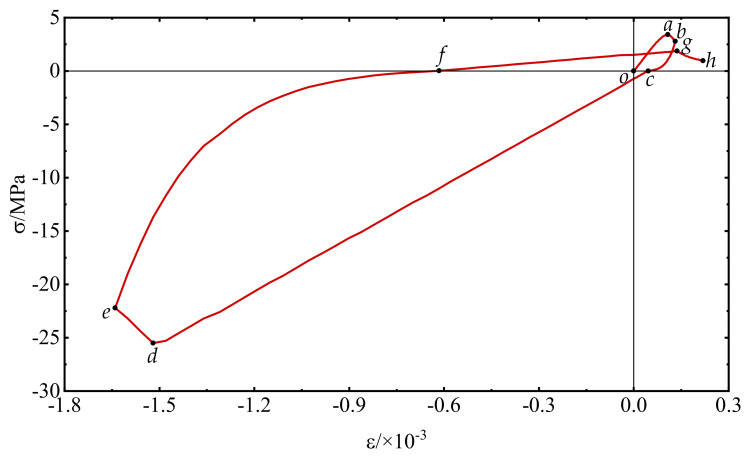
Stress−strain relationship under uniaxial reciprocating loading: the letters *o*, *a*, *b*, *c*, *d*, *e*, *f*, *g*, *h* are points on the curve of the uniaxial reciprocating loading.

**Figure 16:**
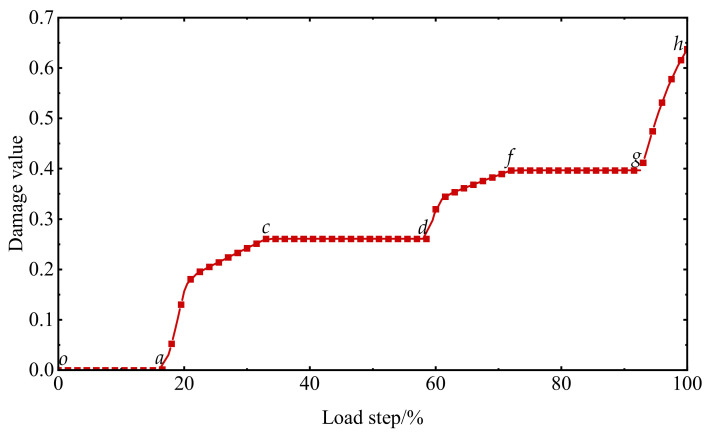
Damage history under uniaxial reciprocating loading: the letters *o*, *a*, *c*, *d*, *f*, *g*, *h* are the positions of the corresponding points in Figure 15 during the damage process.

The stress–strain curve and damage history under reciprocating loading are shown in Figure 15 and Figure 16, respectively. The specimen is subjected to tension at the beginning, for the elastic phase (*o-a*) has not yet caused damage. After the stress value reaching to the uniaxial tensile strength, it turns to the softening phase and then is unloaded at point b to the stress zero-point c. At the same time (*a-b-c*), the damage value of the specimen begins to accumulate gradually. Subsequently, the specimen continues reverse loading along the tangential stiffness direction of the unloading point, and the force state changes from tensile to compressive. During the elastic phase of reverse loading (*c-d*), the damage values remain constant. After reaching the peak compressive stress, the specimen again enters the softening phase (*d-e*), and the damage value continues to increase. Then, the specimen enters the tensile state again after unloading to the stress zero-point f and is loaded for the second time (*f-g-h*).

### 4.5. Koyna Gravity Dam Seismic Conditions

**Figure 17:**
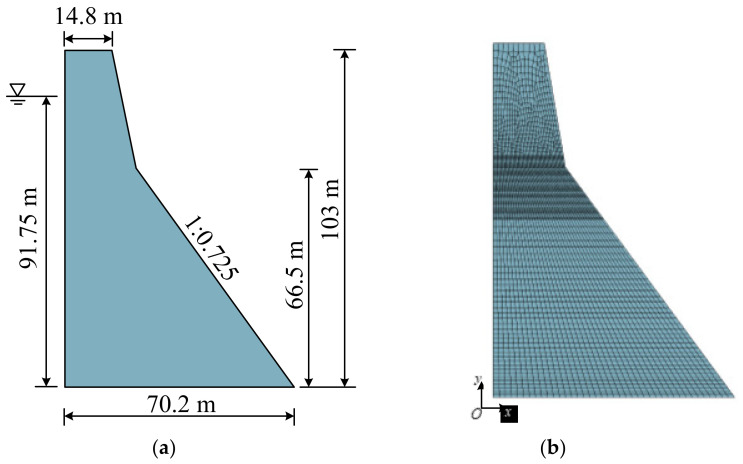
The Koyna dam: (**a**) geometry and dimensions and (**b**) finite element model.

**Figure 18:**
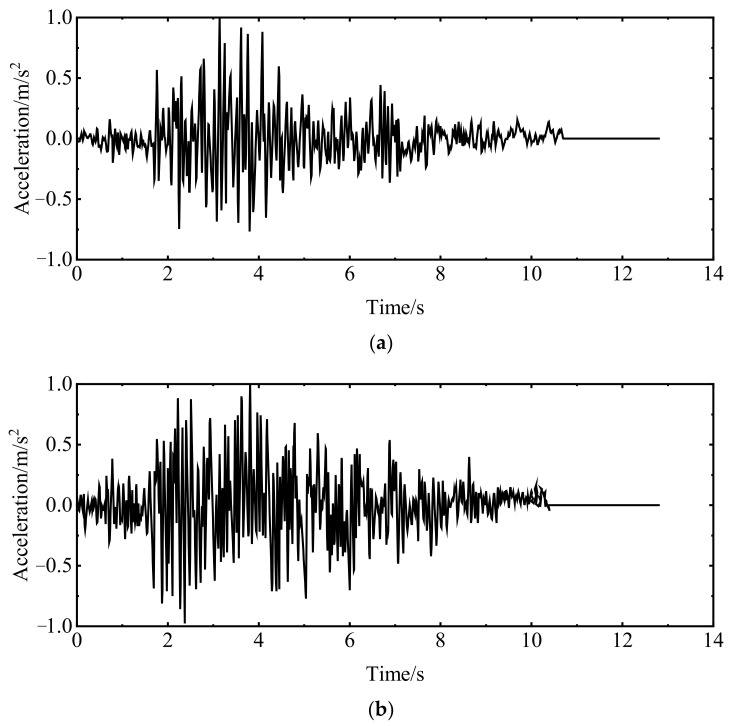
Time history of seismic acceleration: (**a**) horizontal and (**b**) vertical.

The Koyna gravity dam was selected as the research object for numerical verification of the engineering structure. The dam model is shown in Figure 17. The time history method was used for dynamic analysis, and the generalized Newmark method was used to determine the stress distribution and deformation of the dam and foundation at each moment. The fixed artificial boundary was used as the boundary condition of the foundation. That is, in the massless foundation model, a normally fixed constraint was imposed on the truncated side boundary of the foundation, and a two-way fixed constraint was imposed on the bottom boundary. The inertial force was only applied to the dam, and the foundation was only considered for its stiffness. The seismic load was a Koyna seismic wave. The normalized acceleration time history curve is shown in Figure 18. The seismic wave time is 12.8 s, the horizontal peak acceleration is 0.474 g, and the vertical peak acceleration is 0.312 g. The Westgaard additional mass method was used to consider the hydrodynamic pressure of the reservoir water acting on the dam under the seismic loadings.

**Figure 19:**
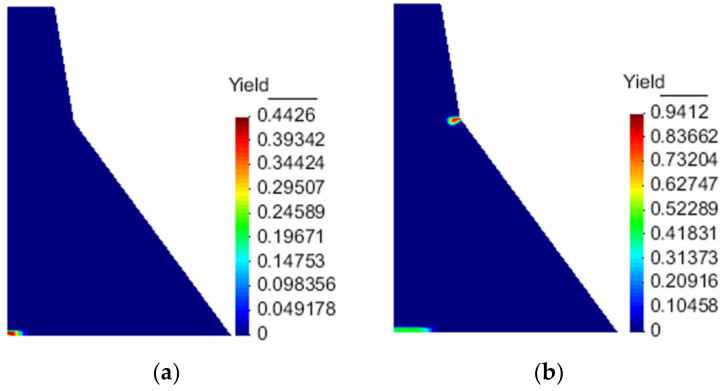

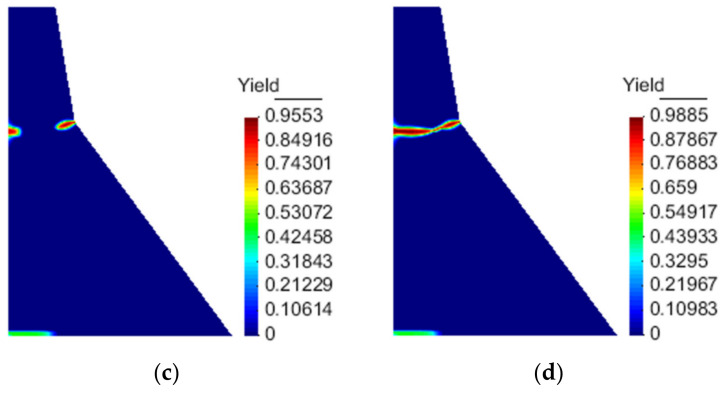
Damage distribution of the Koyna Dam at different times in the simulation results of the proposed model: (**a**) t = 2.7 s; (**b**) t = 4.0 s; (**c**) t = 4.26 s; (**d**) t = 4.5 s.

**Figure 20:**
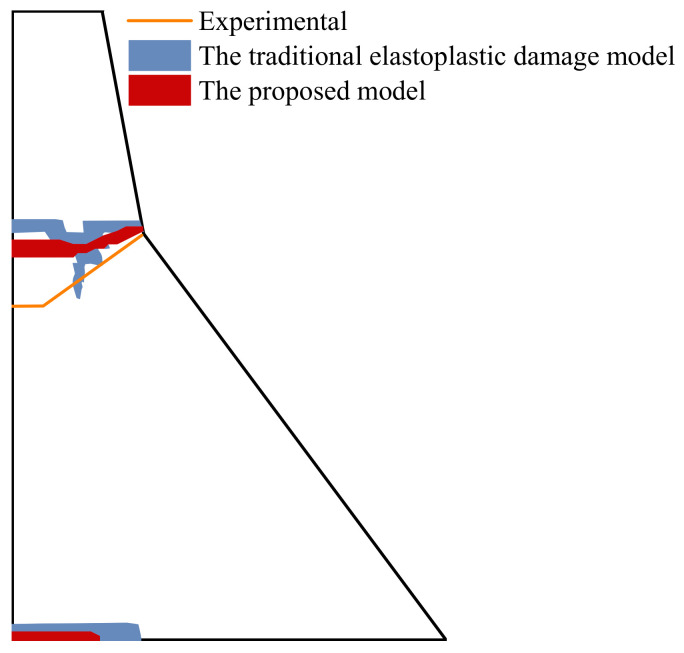
Comparison of the final cracking failure mode.

The damage evolution process is shown in Figure 19. The dam body enters the damage at the corner of the dam slope at about 2.7 s. As it extends to the upstream surface, the upstream surface begins to be damaged and develops to the corner of the dam slope at about 3.4 s, and finally forms through failure. As illustrated in Figure 20, the result calculated by the proposed model is closer to the experimental result [46] than the traditional elastoplastic damage model [47]. After taking into account the concrete hysteresis effect, the damage accumulation during the unloading of the softening section will be considered. Furthermore, the local stress redistribution makes the overall dynamic response result more in line with the actual engineering seismic damage situation.

## 5. Conclusions

Based on the four-parameter damage model of concrete, a concrete damage model considering the hysteresis effect under cyclic loading is established. On the basis of the principle of strain equivalence, the model converts the complex multiaxial force and deformation problem into a simple equivalent strain space for a solution. The damage variable is solved by the uniaxial damage evolution equation with the equivalent effect as independent variables, and then the true stress in the multiaxial state is obtained. The complex nonlinear properties of concrete under cyclic loadings, such as conversion between tensile and compressive, stiffness degradation, strength softening, and irreversible deformation, can be considered. At the same time, hysteresis effects, including the resulting material stiffness degradation and damage accumulation, can be adequately described. Through the comparison with the classical experimental test and the seismic condition of the Koyna gravity dam, the model can well reflect the force state and deformation law of the concrete under the cyclic reciprocating load. The calculation process does not depend on the structure form, loading law, and material parameters. It can provide support for further research on the seismic performance of concrete structures.

## Figures and Tables

**Table 1:** Material parameters of tests.

Test		ρ (kg/m^3^)	E (GPa)	$f_{t}$ (MPa)	υ	$f_{t}/f_{c}$	a	γ
Three-point bending beam		2400	31.6	5.2	0.2	0.10	8.44	--
Uniaxial cyclic tensile load		2400	31.7	3.4	0.17	0.10	3.76	--
Uniaxial cyclic compressive load		2400	22.4	4.0	0.2	0.10	--	1.2
Koyna gravity dam	dam	2643	31.0	2.9	0.2	0.12	2.62	--
	foundation	2700	20.0	--	0.2	--	--	--

## Data Availability

All the data in the tests of this study have been listed in the paper.
